# Strength and Permeability Properties of Pervious Concrete Containing Coal Bottom Ash Aggregates

**DOI:** 10.3390/ma15217847

**Published:** 2022-11-07

**Authors:** Ji-Hun Park, Seung-Tae Jeong, Quang-The Bui, In-Hwan Yang

**Affiliations:** Department of Civil Engineering, Kunsan National University, Gunsan-si 54150, Korea

**Keywords:** compressive strength, compaction level, permeability, coal bottom ash, total void ratio

## Abstract

This study investigates the strength and permeability properties of pervious concrete-containing coal bottom ash (CBA) aggregates. Two pervious concrete mixtures were fabricated with different aggregate size distributions. One mixture contained CBA aggregates with a single-type distribution and the other mixture contained CBA aggregates with a hybrid-type distribution. The test parameters of the CBA pervious concrete included the water/cement (W/C) ratio and compaction level to investigate their effects on the properties. W/C ratios of 0.25, 0.30, and 0.35 were considered for the mixture, and compaction levels of 0.5, 1.5, and 3.0 MPa were applied to fabricate the pervious specimen. The increase in the W/C ratio reduced the strength by approximately 20% to 30% of the CBA pervious concrete. The increase in the compaction level reduced the permeability by approximately four to five times but significantly increased the strength of the CBA pervious concrete. The test results indicate that the use of single-type CBA or hybrid CBA aggregates with different size distributions affected the properties of the pervious concrete. The strength of specimens, including hybrid CBA aggregates, was 30% to 45% greater than that of the specimens containing single-type CBA aggregates. Meanwhile, the use of hybrid CBA aggregates reduced the permeability of the CBA pervious concrete by approximately 20% to 35%. Finally, relationships between the strength properties, permeability characteristics and total void ratios of the CBA pervious concrete specimens are suggested based on the test results.

## 1. Introduction

The demand for electricity has been increasing over time, which is motivating the development of thermal power plants. According to a study by Abbas et al. [1], the amount of coal bottom ash (CBA) emitted from thermal plants worldwide exceeded 700 million tons in one year. Moreover, over 9 million tons of CBA are generated in South Korea every year [2]. This indicates that CBA waste from thermal power plants should be utilized to minimize the influence of CBA on the environment. Several studies have noted that CBA can be used as a concrete component [3,4,5,6,7]. It is recommended that CBA should be used for lightweight or pervious concrete [4,8] because of its porous structure.

For pervious concrete, coarse aggregates and cement paste act as the skeleton; thus, in the usage of coarse aggregates, the type and size might directly affect the properties of the concrete. In a study by Ćosić et al. [9], it was found that the use of small aggregates improved the density, which resulted in a higher strength of the pervious concrete. Strieder et al. [10] reviewed the use of recycled coarse aggregates with different sizes and proportions; they fabricated pervious concrete with various replacement levels of natural coarse aggregates by recycled coarse aggregates. According to this study, the density of the pervious concrete decreased with increasing coarse aggregate size. In addition, the permeability of the pervious concrete increased as the size of the coarse aggregate increased. Park et al. [11] investigated the feasibility and strength properties of pervious concrete-containing CBA of different sizes. Their test results showed that the permeability of the pervious concrete containing larger CBA aggregates was greater than that with smaller CBA aggregates. In addition, the compressive and flexural tensile strengths of the pervious concrete decreased with the larger size of the CBA.

Kováč and Sičáková [12] investigated the strength properties of pervious concrete fabricated with a W/C ratio ranging from 0.25 to 0.35. It was found that an insignificant difference in strength properties occurred when the W/C ratio increased from 0.25 to 0.35. However, the influences of the W/C ratio on the strength properties of pervious concrete were presented in research by Li et al. [13]. The researchers concluded that the W/C ratio affected the paste film thickness, which played a major role in the permeability and strength of pervious concrete. To achieve the desired porosity, Costa et al. [14] fabricated pervious concrete with different W/C ratios. The researchers reported that the strength properties of the pervious concrete somewhat depended on the W/C ratios, but the permeability was highly affected by the W/C ratios. Therefore, the test results in previous studies implied that the strength properties of pervious concrete mainly depended on the contents of the cement paste and aggregates; accordingly, the W/C ratio affected its properties.

The workability of pervious concrete is lower than that of normal concrete due to the concrete mixture containing coarse aggregate and cement paste. The lack of fine aggregates reduces the workability of pervious concrete, potentially decreasing the adhesion between the coarse aggregates and the cement paste. Therefore, precompaction is necessary when casting pervious concrete. It has been reported that pervious concrete mixtures under different compaction levels show different properties [15].

However, most previous studies have focused on analyzing the strength and permeability of pervious concrete containing only natural aggregates. There are few studies on the strength and permeability of pervious concrete-containing CBA aggregates.

Therefore, this study investigates the permeability and strength properties of CBA pervious concrete, including the compressive, splitting, and flexural tensile strengths. The effects of the size distributions of CBA aggregates, W/C ratios, and compaction levels on the properties of CBA pervious concrete are evaluated. Finally, the relationships between the strength, permeability, and total void ratio are presented to obtain prediction equations for the strength and permeability of CBA pervious concrete.

## 2. Experimental Details

**Figure 1:**
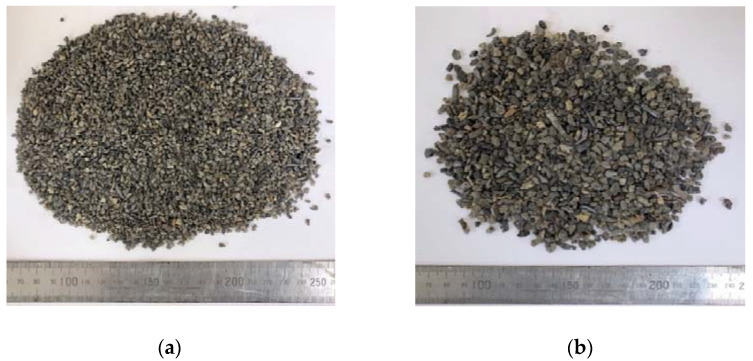
CBA used: (**a**) 1.2~2.5 mm; (**b**) 2.5~5.0 mm.

The single and hybrid CBAs used in the mixtures were provided by a thermal power plant company in Yeongheung, Korea. According to KS F 2504 [16], density and absorption rate tests for CBA were performed, and CBA aggregate with a weight of 150 g was tested under saturated-surface dry (SSD) conditions. The density and absorption rate tests of the CBA were mainly performed on three different aggregate series. First, the CBA was divided into two groups with aggregate sizes of 1.2–2.5 mm and 2.5–5.0 mm; from there, the density and absorption rate experiments were performed. Finally, density and absorption rate tests were conducted on composite aggregates in which 20% of aggregates 1.2–2.5 mm in size and 80% of aggregates 2.5–5.0 mm in size were combined by volume. The CBA aggregates used in the mixtures are presented in Figure 1.

**Figure 2:**
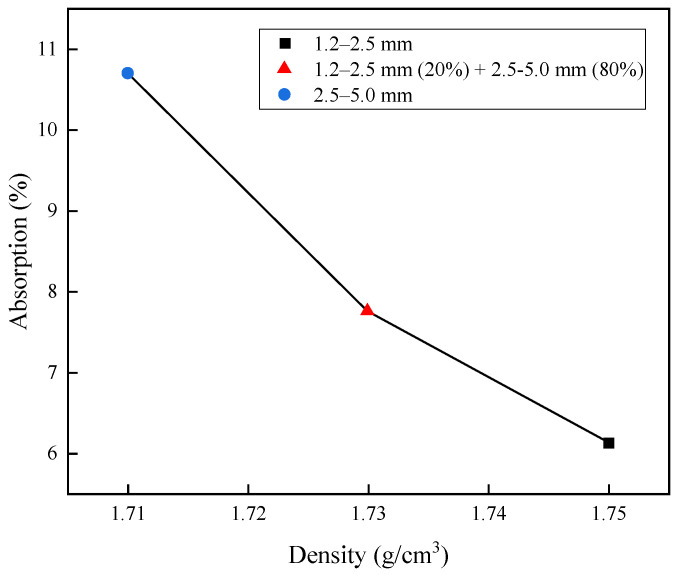
Relationship between absorption and density of CBA types.

Table 1 shows the density and water absorption test results according to the size of the CBA, and Figure 2 illustrates the aggregate density and absorption rate test results at different sizes of CBA aggregates. The SSD density of the CBA aggregate ranged between 1.71 and 1.75, which was in accordance with previous studies [17,18,19,20,21] that satisfied the standard bottom ash density in a range from 1.0 to 2.0. Water absorption for each size of bottom ash ranged from 6.13 to 10.7%. As shown in Figure 2, the density increased with decreasing CBA size, and water absorption decreased. Since CBA is a porous material with many pores, it was indicated that the lower the density was due to the porosity, the higher the water absorption through the pores [22].

Ordinary Portland cement (OPC) type I, achieving 51 MPa in compressive strength after 28 days, was used for the mixtures. The cement was supplied by the Ssangyong C & E Company (Seoul, Korea). The OPC had a density and specific surface area of 3.13 g/cm^3^ and 3670 cm^2^/g, respectively.

## 3. Experiment

### 3.1. Mixing Proportions

Two series of mixtures, as shown in Table 2, were prepared in terms of CBA types with different sizes. For the first mixture series, a single CBA aggregate with sizes of 2.5~5.0 mm was applied. For the second mixture series, particles with sizes of 1.2~2.5 mm and particles with sizes of 2.5~5.0 mm were combined with a proportion of 20% and 80% by volume. The letters “S” and “H” in the mixture names denote the CBA types used in the mixtures. In the mixture identifications in Table 2, the two numbers following “W” indicate W/C ratios of 0.25, 0.30, and 0.35. The digit numbers following “C” indicate compaction levels of 0.5, 1.5, and 3.0 MPa.

The CBA aggregates were prepared under SSD conditions before they were mixed in concrete because of their high-water absorption capacities. Finally, to improve the adhesion between CBA aggregates, a cohesive agent was used in the mixtures. The cohesive agent was a viscosity-modifying admixture, including water-soluble polymers that improved the viscosity of the mixing water. In addition, these water-soluble polymers in cohesive agents retain cement constituents in suspension, so the adhesion between CBA aggregates was increased.

### 3.2. Compaction Method

**Figure 3:**
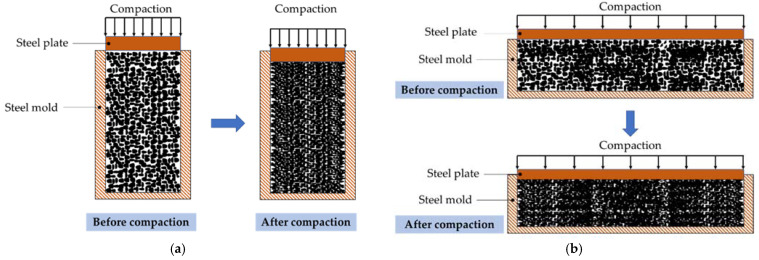
Schematic compacting method. (**a**) Compaction on a cylindrical specimen; (**b**) compaction on a prismatic specimen.

Figure 3 illustrates the process of the compaction method in cylindrical and prismatic steel molds after the pervious concrete was mixed and poured into the molds. The cylindrical mold had dimensions of 100 mm (diameter) × 230 mm (height) × 10 mm (thickness), while the dimensions of the prismatic mold were 100 mm (width) × 130 mm (height) × 400 mm (length) × 10 mm (thickness). After pouring the fresh CBA concrete into the steel mold, a steel plate was placed on the upper surface of the specimen. As shown in the figure, the specimen was compacted by the steel plate, and a corresponding load was applied by using a hydraulic jack.

**Figure 4:**
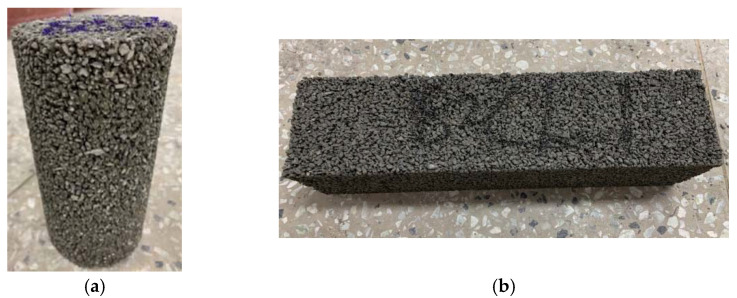
Compacted CBA pervious concrete specimens. (**a**) Cylindrical specimen; (**b**) prismatic specimen.

Since the concrete had not yet hardened in the fresh stage after mixing, the cohesive strength between the cement paste and the aggregates was weak. This cohesive strength could be improved by applying a precompaction load. However, excessive compaction could result in segregated CBA aggregates in fresh concrete. Hence, the compaction load was limited, and three levels of compaction (0.5, 1.5, and 3.0 MPa) were considered. The compacted CBA pervious specimens are shown in Figure 4. The surfaces of the cylindrical and prismatic specimens indicated that the CBA pervious specimen became denser after compaction.

### 3.3. Measurement of Material Properties

The material properties, including the total void ratio, permeability, and strength, of the CBA pervious concrete were examined after the concrete specimens were cured in water at 24 ± 2 °C for 28 days.

The total void ratio of the CBA pervious specimens was calculated as follows:

(1) $$T(\%)=1-\left(\frac{W_{d}-W_{w}}{\rho_{w}\times V_{c}}\right)\times 100$$

where *T* (%) is the total void ratio, *W_d_* (g) is the mass of the CBA pervious concrete in the dry state, *W_w_* (g) is the mass of the CBA pervious concrete weighed in water, *ρ_w_* (g/cm^3^) is the density of water, and *V_c_* (cm^3^) is the cylindrical concrete specimen volume.

**Figure 5:**
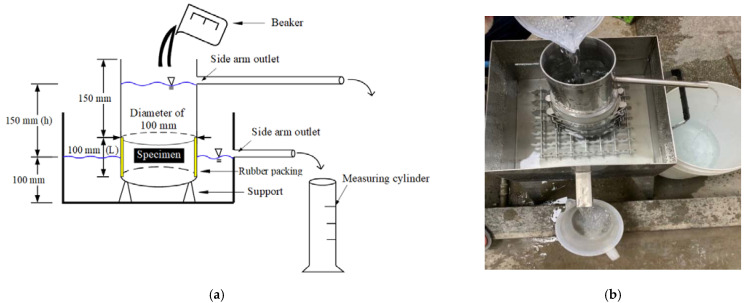
Permeability test setup. (**a**) Schematic of the permeability test; (**b**) performance of the permeability test.

Figure 5 shows the measurement of the permeability of the CBA pervious concrete. A concrete specimen with dimensions of 100 mm (height) 100 mm (diameter) was prepared and fixed in a steel cylinder. The tested specimen was cut from the cylindrical specimens with dimensions of 100 mm (height) × 200 mm (diameter). Then, the steel cylinder was placed on a steel grid in a steel box to ensure water drained through the CBA pervious specimen. The water was poured on top of the pervious concrete specimen for 30 s. The water depth remained constant at 100 mm in the steel box. The water draining through the specimen increased the amount of water in the steel box. When the water depth in the steel box reached 100 mm, the water flowed through the side arm outlet of the steel box into the measuring cylinder. According to the volume of water in the measuring cylinder, the coefficient of water permeability of the CBA pervious concrete was calculated as follows:

(2) $$T_{\mathrm{coef}}=\frac{L}{h}\times \frac{Q}{A\times (t_{2}-t_{1})}$$

where *T*_coef_ (mm/s) is the coefficient of water permeability, *L* (mm) is the height of specimen, *h* (mm) is the difference in water head between the steel cylinder and steel box, *Q* (mm^3^) is the volume of water in the measuring cylinder, *A* (mm^2^) is the cross-section area of the specimen, and *t*_1_ and *t*_2_ (s) are the initial and final times of the flowing water from the side arm outlet of the steel box. The height of the water pressure through the sample or the constant water head difference between the steel cylinder and steel box (*h*) was estimated from the water head of the bottom of the side arm outlet in the water box to the water head of the bottom of the side arm outlet in the water cylinder.

The compressive strength of the CBA pervious concrete was estimated according to the European standard EN 12390-3:2019 [23]. Moreover, the splitting tensile strength of the CBA pervious concrete was determined according to the European standard EN 12390-6:2009 [24]. For the flexural tensile test, the experiment was performed according to the European standard EN 12390-5:2019 [25].

## 4. Test Results and Discussion

### 4.1. Total Void Ratio

**Figure 6:**
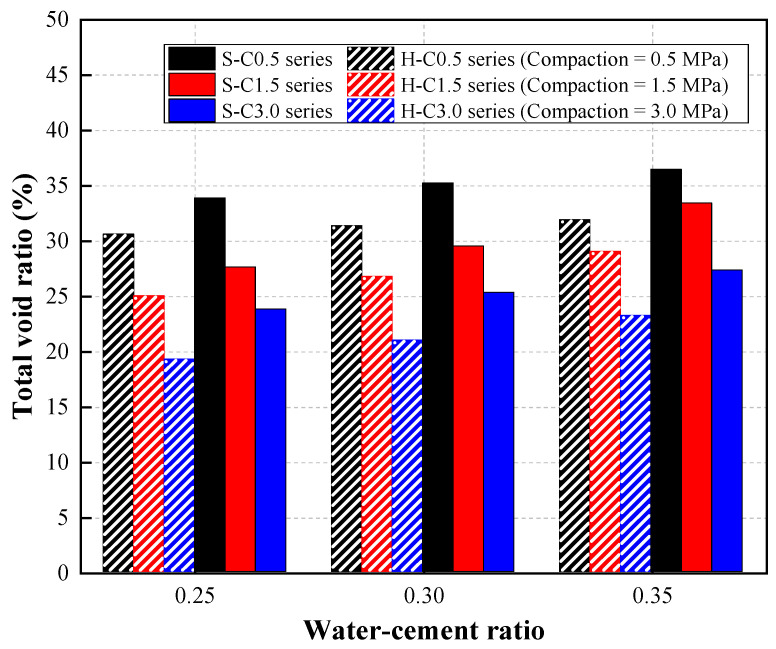
Total void ratio results.

The test results of the total void ratio of CBA pervious concrete at various W/C ratios are shown in Table 3 and illustrated in Figure 6.

The test results reveal that the total void ratios of both the single-type and hybrid CBA aggregate mixture series increased as the W/C ratio increased. For the mixture series containing single-type CBA aggregates (S mixture series), an increase in the total void ratio was observed with increasing W/C ratios. Specifically, for the S-C0.5 mixture specimens, the total void ratio increased by 3.99% and 7.65% as the W/C ratio increased from 0.25 to 0.30 and 0.35, respectively. Similarly, the total void ratios of the S-W25-C1.5, S-W30-C1.5, and S-W35-C1.5 specimens were 27.6%, 29.5%, and 33.4%, respectively, at each W/C ratio. For the S-C3.0 mixture series, the total void ratio of the S-W35-C3.0 specimen reached its greatest value of 27.3%, while those of the S-W25-C3.0 and S-W30-C3.0 specimens were 23.8% and 25.3%, respectively.

For the mixture series containing hybrid CBA aggregates (H mixture series), the value of the total void ratio of the H-C0.5 series specimens was slightly increased by increasing the W/C ratio from 0.25 to 0.35. An increasing tendency in the total void ratio was observed in the H-C1.5 series specimens. The total void ratio of the H-W25-C1.5 specimen was 25.0%, while those of the H-W30-C1.5 and H-W35-C1.5 specimens were 26.8% and 29.0%, respectively. W/C ratios of 0.30 and 0.35 led to increases in the total void ratios of the H-C3.0 series specimens by 8.88% and 20.62%, respectively.

According to previous studies [14,26,27,28], the total void ratio of the pervious concrete increased with increasing W/C ratio. In particular, Cui et al. [28] investigated the effects of W/C ratios ranging from 0.32 to 0.40 on the properties of pervious concrete. The test results in their study indicated that the total void ratio slightly increased with increasing W/C ratio. This phenomenon occurred because the higher W/C ratio in the mixture resulted in shrinkage in the cement paste, thus creating more microscopic pores in the concrete.

This figure also presents the effects of the compaction levels on the total void ratios of CBA pervious concrete specimens. The total void ratio of the S-W25-C3.0 specimen with a 0.25 W/C ratio was 29.79% and 13.77% lower than that of the S-W25-C0.5 and S-W25-C1.5 specimens, respectively. The S-W30-C3.0 specimen exhibited the lowest total void ratio of 25.3%, while those of the S-W30-C0.5 and S-W30-C1.5 specimens were 35.2% and 29.5%, respectively. Similarly, the total void ratio of the S-W35 mixture series decreased from 36.5% to 27.3% as the compaction levels increased from 0.5 to 3.0 MPa.

For the concrete specimens containing hybrid CBA aggregates, the total void ratio of the H-W25 series was reduced by 18.28% and 37.05% at compaction levels of 1.5 and 3.0 MPa, respectively. Similarly, the H-W35-C3.0 specimen showed the lowest total void ratio of 23.2%, while those of the H-W35-C0.5 and H-W35-C1.5 specimens were 31.9% and 29.0%, respectively.

Applying compaction to CBA pervious concrete caused the concrete to become denser since the distances between aggregates decreased, and thus the cement paste was thicker [29] in the horizontal direction. When applying the compaction to CBA pervious concrete vertically, the distance between particles was reduced, and the coating cement paste of the particles was increased horizontally. Simultaneously, the excess cement paste filled the voids between the particles. According to a previous study, Torres et al. [30] measured the cementitious paste thickness of uncompacted and compacted pervious concrete in the horizontal direction. The test results of this study showed that the horizontal thickness of the cementitious paste of the compacted specimens was higher than that of the uncompacted specimens. In another previous study by Wang et al. [31], the cementitious thickness of pervious concrete was measured by high-precision CT scanner equipment. It was reported that the bonding region of the coating cementitious paste in the horizontal direction played an important role in the strength and permeability of the pervious concrete. The thickness of the bonding region of the cementitious paste coating increased the strength of the pervious concrete but reduced its permeability. Therefore, the total void ratio was decreased due to the compaction of the pervious concrete.

**Figure 7:**
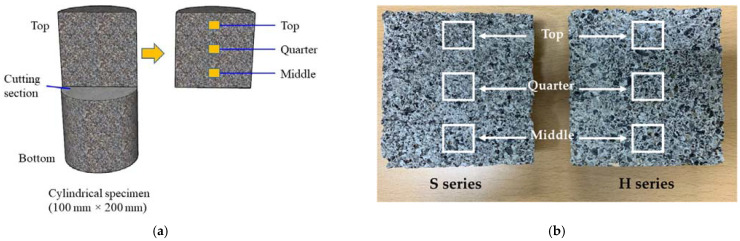
Extraction method. (**a**) Illustration of the extraction method; (**b**) extraction positions in two series.

**Figure 8:**
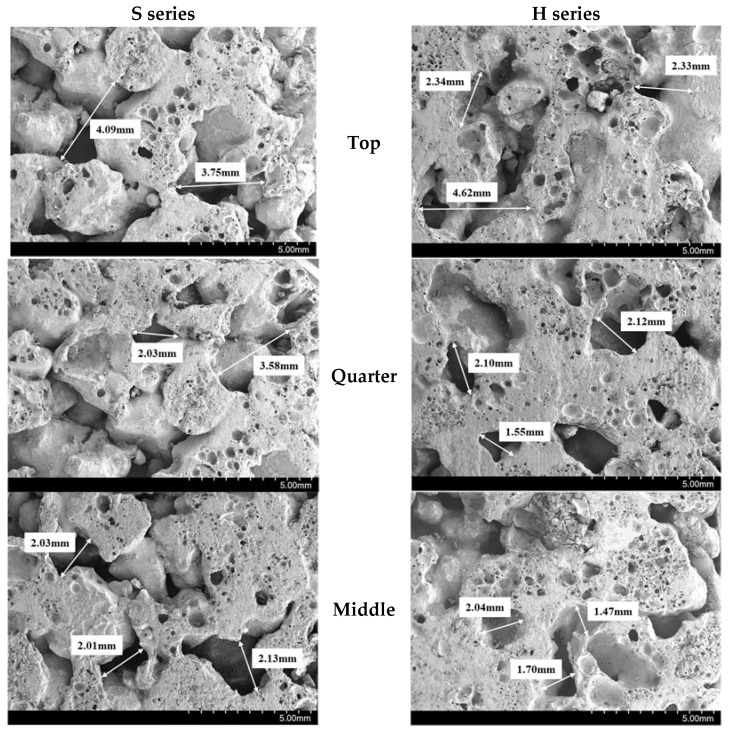
SEM results.

Moreover, the total void ratio of the pervious concrete with single-type CBA aggregates was higher than that of the pervious concrete with hybrid CBA aggregates. To prove this claim, a scanning electron microscopy (SEM) analysis was conducted. Two cylindrical specimens of each mixture with single-type and hybrid CBA aggregates were cut into halves. Then, as presented in Figure 7, samples (dimensions of 20 mm × 20 mm × 10 mm) were extracted from three different locations of the upper half cylinder over the depth of the cylindrical concrete specimen. As presented in Figure 8, the distances between the particles of the specimen with hybrid CBA aggregates (H series mixture) were smaller than those of the specimen with single-type CBA aggregates (S series mixture) at three different positions. This finding implied that the use of CBA types affected the microstructures of the CBA pervious concrete specimens. Therefore, microstructure analyses of the CBA pervious concrete specimens with SEM images corresponded to the test results showing that the total void ratio of the pervious concrete with hybrid types of CBA aggregates was lower than that of the pervious concrete with single-type CBA aggregates.

In addition, the SEM results implied that the compaction had a different influence on the voids at each position in the pervious concrete specimen. This finding shows that the voids in CBA pervious concrete or the distances between the aggregate particles decreased from the top to the middle position of the specimens in both series. This phenomenon implied that the cohesive strength between the CBA aggregates was enhanced by compacting the aggregates. Nevertheless, when the compaction energy was released, the compacted particles expanded to obtain the initial stage. Therefore, the microstructure at the middle position was denser than that at the top position.

### 4.2. Permeability

**Figure 9:**
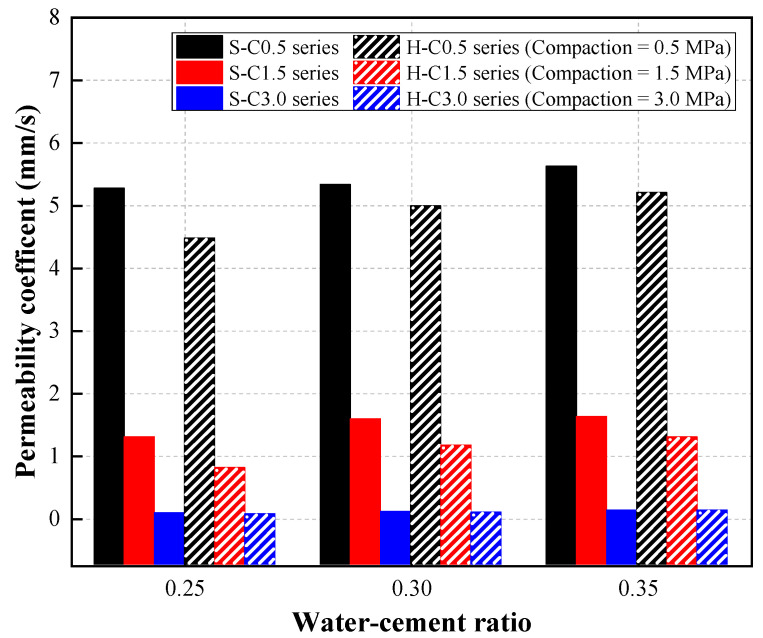
Permeability coefficient results.

Figure 9 shows the permeability test results of CBA pervious concrete. The results showed the influences of the W/C ratio on the permeability of the CBA pervious concrete. Specifically, for the S mixture, the permeability coefficient of the S-C0.5 specimen increased from 5.27 mm/s to 5.62 mm/s as the W/C ratio increased from 0.25 to 0.35. A similar tendency was observed in the S-C1.5 mixture series specimens with permeability coefficients of 1.29, 1.58, and 1.62 mm/s at W/C ratios of 0.25, 0.30, and 0.35, respectively. However, the permeability coefficient of the S-C3.0 mixture series was almost constant at various W/C ratios.

A slight increasing tendency in permeability was observed in the H mixture series. For the H-C0.5 mixture series, the permeability coefficient increased by 11.63% and 16.33% as the W/C ratio increased from 0.25 to 0.30 and 0.35, respectively. For the H-C1.5 mixture series, the H-W25-C1.5, H-W30-C1.5, and H-W35-C1.5 specimens were 0.80, 1.16, and 1.29 mm/s at each W/C ratio, respectively. As in the case of the S-C3.0 series, the permeability of the H-C3.0 mixture series was constant at various W/C ratios.

The test results in this study show that the permeability of the CBA pervious concrete slightly increased with increasing W/C ratio. In particular, when the compaction level was 3.0 MPa, the permeability of the concrete was not affected by W/C ratio. Cui et al. [28] reported that the W/C ratio had little influence on the permeability of pervious concrete. The researchers concluded that the permeability of pervious concrete was mainly related to the porosity of the concrete. The amount of cement paste decreased with increasing W/C ratio, and thus, the porosity of the pervious concrete increased. Eventually, the permeability of pervious concrete increased with increasing W/C ratio.

As shown in Figure 9, the compaction levels had a significant effect on the permeability of the CBA pervious concrete. The test results show that the permeability of the S-W25 mixture series was dramatically reduced by 75.52% with increasing the compaction level to 1.5 MPa. Similarly, the permeability coefficients of the S-W30 and S-W35 mixture series significantly decreased by 70.36% and 71.17%, respectively. Increasing the compaction level of CBA pervious concrete from 1.5 to 3.0 MPa reduced its permeability dramatically. It was recognized that the permeability coefficients of the S-C1.5 mixture series were approximately reduced by 95% at various W/C ratios.

For the H mixture series, a similar decreasing tendency in the permeability affected by the compaction level was recognized. The permeability of the S mixture series significantly decreased by approximately 95% at various W/C ratios with increasing compaction level. In particular, when a compaction level of 3.0 MPa was applied to the specimens, the H mixture series almost lost its permeability. Therefore, low permeability coefficients of nearly 0 mm/s of the H mixture series were obtained at various W/C ratios.

In addition, the test results indicate that the permeability coefficients of the S mixture series were greater than those of the H mixture series at various W/C ratios. As shown in Figure 6, the H mixture series microstructures were denser than the S mixture because of the size distributions of coarse aggregates. Adding the small aggregate to the mixture could fill the voids between the larger aggregates, preventing the flow rate of water through the pervious concrete. Therefore, a lower permeability was shown in the H mixture series.

### 4.3. Compressive Strength of CBA Pervious Concrete

**Figure 10:**
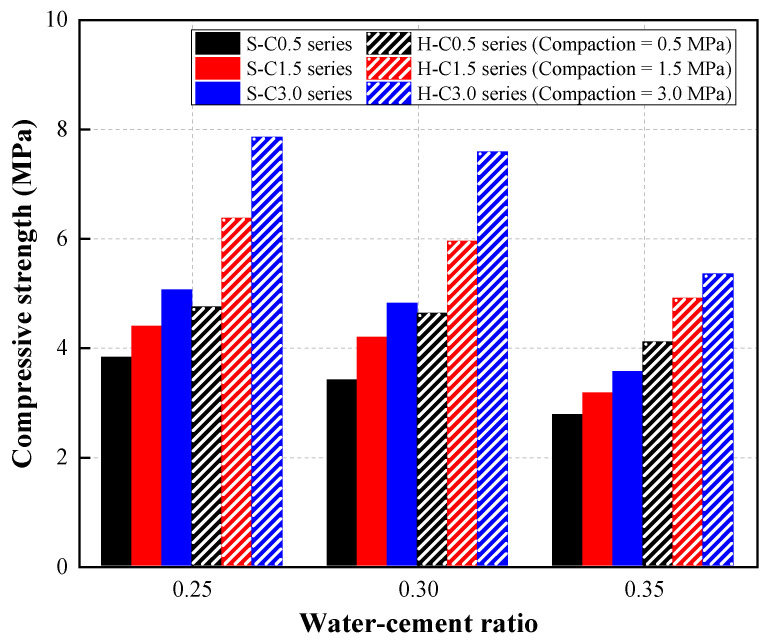
Compressive strength results.

The compressive strength of CBA pervious concrete is shown in Figure 10. The figure shows that the compressive strength of the CBA pervious concrete decreased as the W/C ratio increased in both the single-type and hybrid CBA mixture series. For the hybrid CBA mixture series, the compressive strength gradually decreased with increasing W/C ratio. A decreasing tendency in compressive strength with the W/C ratio was observed in the single-type CBA mixture series. Specifically, it was shown that the compressive strength of the single-type CBA mixture under a compaction of 0.5 MPa (S-C0.5) decreased from 3.82 to 2.77 MPa as the W/C ratio increased from 0.25 to 0.35. Additionally, the compressive strengths of the S-C1.5 and S-C3.0 mixture series decreased significantly with increasing W/C ratio.

The strength at the interfacial transition zone (ITZ) between CBA aggregates and cement paste played an important role in the strength of the concrete. An increase in the W/C ratio could generate micropores around the ITZ and finally reduce the strength of the CBA pervious concrete. In addition, according to previous studies [14,28,32], the increase in the W/C ratio caused the cement paste to be more liquefied; therefore, the cement pastes easily flowed on the faces of the aggregates from the upper part to the bottom part of the specimens. In this way, the strength of the upper part of the pervious specimen was lower than that of the bottom part, which reduced the strength of the whole specimen.

The effect of the compaction level on the compressive strength of CBA pervious concrete can be found in this figure. The test results show that the compaction level improved the compressive strengths of both mixture series.

For the pervious concrete specimens with hybrid types of CBA aggregates, the influence of the compaction level on the compressive strength of the pervious concrete specimens was considerable. As an example, the compressive strengths of concrete specimens with compactions of 1.5 (H-W25-C1.5) and 3.0 MPa (H-W25-C3.0) were 34.39% and 65.61% greater than those of the concrete specimens with a compaction of 0.5 MPa (H-W25-C0.5) at a W/C ratio of 0.25.

It was assumed that the cement paste thickness of the CBA pervious concrete was improved by increasing the compaction level. Moreover, the porosity of the CBA pervious concrete decreased, which improved its compressive strength. Sahdeo et al. [33] recognized that the compressive strength of pervious concrete improved with increasing compaction energy. The test results reported in a study by Bonicelli et al. [15] indicated that the porosity of the pervious concrete decreased as the compaction level increased.

The compressive strength of the pervious concrete with hybrid CBA aggregates was greater than that of the pervious concrete with single-type CBA aggregates at various W/C ratios. The denser microstructure of pervious concrete with hybrid CBA aggregates resulted in a higher compressive strength than that of pervious concrete with a single type of CBA aggregate. The same tendency was reported in a study by Yang et al. [34]. This study indicated that the concrete mixture including multiple-size aggregates was more easily compacted than the concrete mixture containing single-size aggregates. Therefore, the strength of the pervious concrete was enhanced by combining different size distributions of the CBA aggregate.

### 4.4. Splitting Tensile Strength of the CBA Pervious Concrete

**Figure 11:**
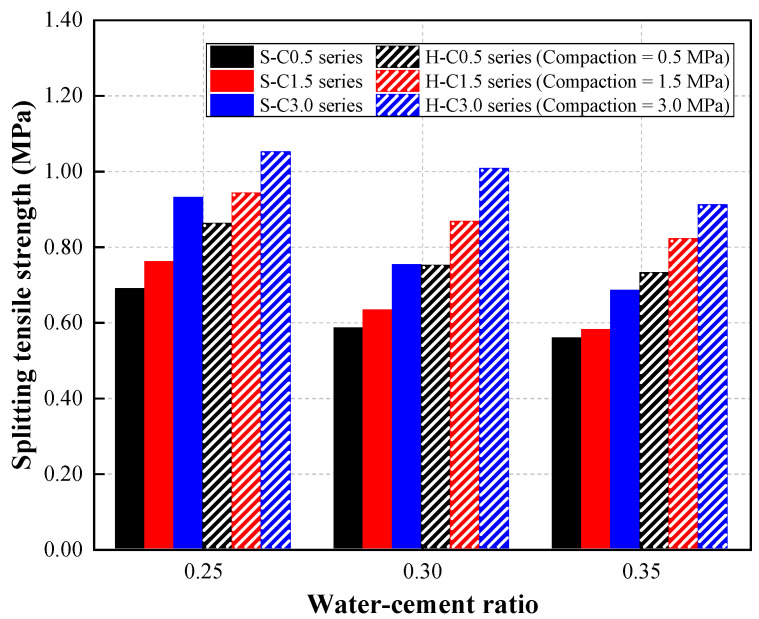
Splitting tensile strength results.

The splitting tensile strength of CBA pervious concrete is shown in Figure 11. The test results reveal that the splitting tensile strength of the CBA concrete decreased with increasing W/C ratio. For a single type of CBA aggregate, the splitting tensile strength of the S-C0.5 mixture series decreased from 0.69 to 0.56 MPa with increasing W/C ratio. The splitting tensile strength of the S-C1.5 mixture series specimens gradually decreased by 17.11% and 23.68% and that of the S-C3.0 mixture series specimen decreased by 19.35% and 26.88% when the W/C ratio increased from 0.25 to 0.30 and 0.35, respectively.

The test results show that the splitting tensile strength of the CBA pervious concrete was improved by applying compaction to pervious CBA concrete. Specifically, for concrete specimens with hybrid CBA aggregates at a W/C ratio of 0.25, the splitting tensile strengths of the H-W25-C0.5, H-W25-C1.5, and H-W25-C3.0 specimens were 0.86, 0.94, and 1.05 MPa, respectively. The splitting tensile strength of concrete specimens with hybrid CBA aggregates at a W/C ratio of 0.30 increased from 0.75 to 1.01 MPa with increasing the compaction level from 0.5 to 3.0 MPa.

A similar tendency of splitting tensile strength in compacted pervious concrete according to the compaction levels was observed in the study by Bonicelli et al. [15]. This study showed that the splitting tensile strength of the pervious concrete improved with an increasing number of blows. By increasing the compaction energy, the aggregate particles were tightly packed, and the cement paste thickness increased. Thus, the strength of the pervious concrete was improved at high compaction levels.

### 4.5. Flexural Tensile Strength of the CBA Pervious Concrete

**Figure 12:**
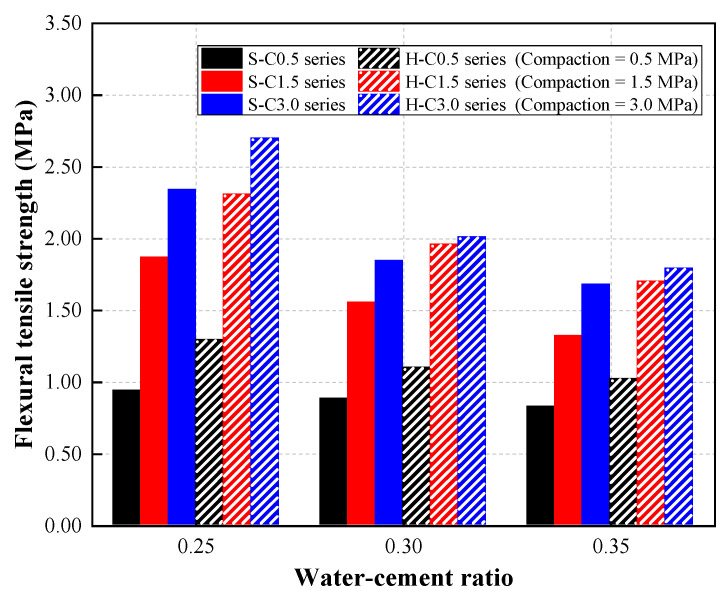
Flexural tensile strength results.

Figure 12 illustrates the flexural tensile strength characteristics of CBA pervious concrete at various W/C ratios and compaction levels for concrete mixtures with single-type and hybrid CBA aggregates.

Overall, the flexural tensile strength of CBA pervious concrete decreased as the W/C ratio increased. For the pervious concrete with single-type CBA aggregates at a compaction level of 0.5 MPa (S-C0.5 series), the flexural tensile strength decreased slightly with increasing W/C ratio. Specifically, the flexural tensile strength of the concrete specimen with a single type of CBA aggregate at a compaction level of 0.5 MPa slightly decreased from 0.94 to 0.82 MPa as the W/C ratio increased from 0.25 to 0.35. Costa et al. [14] investigated the effects of the W/C ratio and admixture on the properties of pervious concrete. Their study indicated that the W/C ratio insignificantly affected the flexural tensile strength of the pervious concrete.

However, the concrete specimen with a single type of CBA aggregate at compaction levels of 1.5 and 3.0 MPa (S-C1.5 and S-C3.0 series) represented a significant reduction in the flexural tensile strength. Specifically, the flexural tensile strength of the concrete specimen with a single type of CBA aggregate at a compaction level of 1.5 MPa decreased by 20.11% and 41.53% as the W/C ratio increased from 0.25 to 0.30 and 0.35, respectively. Therefore, the test results in this study imply that the compaction level of the CBA pervious concrete could be considered when the extent of the effect of the W/C ratio on the flexural tensile strength in the concrete was evaluated.

Regarding the compaction effect, the increase in compaction significantly improved the flexural tensile strength in both mixture series. As shown in this figure, a significant improvement in the flexural tensile strength was observed in pervious concrete with hybrid CBA aggregates due to the compaction levels. The flexural tensile strength of the H-W25-C3.0 specimen was 2.2 times and 1.2 times greater than that of the H-W25-C0.5 and H-W25-C1.5 specimens, respectively. At a W/C ratio of 0.30, the H-W30-C3.0 specimen presented the greatest flexural tensile strength of 2.01 MPa, while those of the H-W30-C0.5 and H-W30-C1.5 specimens were 1.10 and 1.96 MPa, respectively. Similarly, the flexural tensile strengths of the H-W35-C0.5, H-W35-C1.5, and H-W35-C3.0 specimens were 1.02, 1.70, 1.79 MPa, respectively.

Finally, the test results demonstrate that the flexural tensile strength of the CBA pervious concrete was improved by using the hybrid CBA type. This phenomenon was reported in a study by Ćosić et al. [9]. According to this study, the addition of the small aggregate improved the density of the pervious concrete, which resulted in higher flexural tensile strength. A study by Nguyen et al. [35] indicated that the cement paste thickness in different aggregate sizes varied. Because of the overall surface area, the cement paste thickness of the large aggregates could be lower than that of the small aggregates. Therefore, it could be assumed that the strength was improved by adding the small aggregates to the pervious concrete.

## 5. Relationships between Test Results

The total void ratio of CBA pervious concrete is an important parameter used to characterize the property of the concrete. Therefore, relationships used to predict the compressive and splitting tensile strength and permeability coefficient of the CBA pervious concrete based on the total void ratio were analyzed.

**Figure 13:**
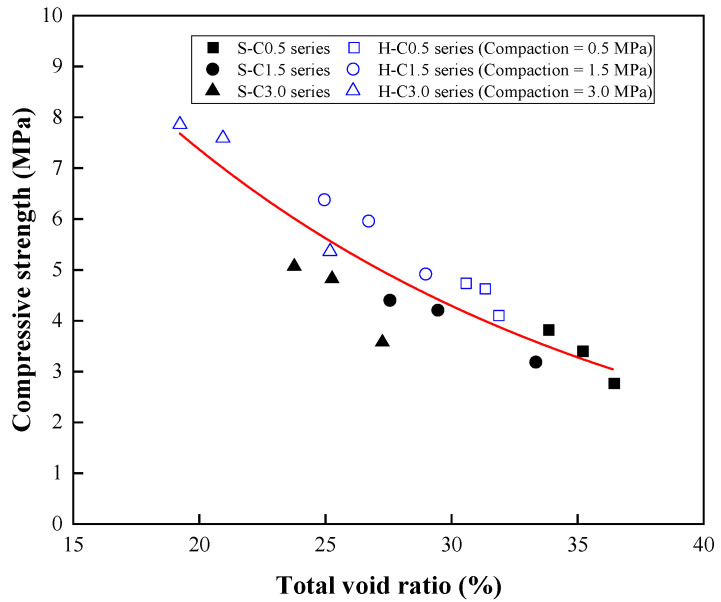
Relationship between compressive strength and total void ratio.

The relationship between the compressive strength and the total void ratio of CBA pervious concrete is illustrated in Figure 13. The relationship revealed that the compressive strength of the CBA pervious concrete decreased as the total void ratio increased. Based on the exponential regression analysis, the relationship between the compressive and total void ratio was expressed by the following equation:

(3) $$f_{c}^{\prime }=21.7856e^{-0.0542v}\;\;\;R^{2}=0.79$$

where *f_c_*′ is the compressive strength (MPa) and *ν* is the total void ratio (%).

**Figure 14:**
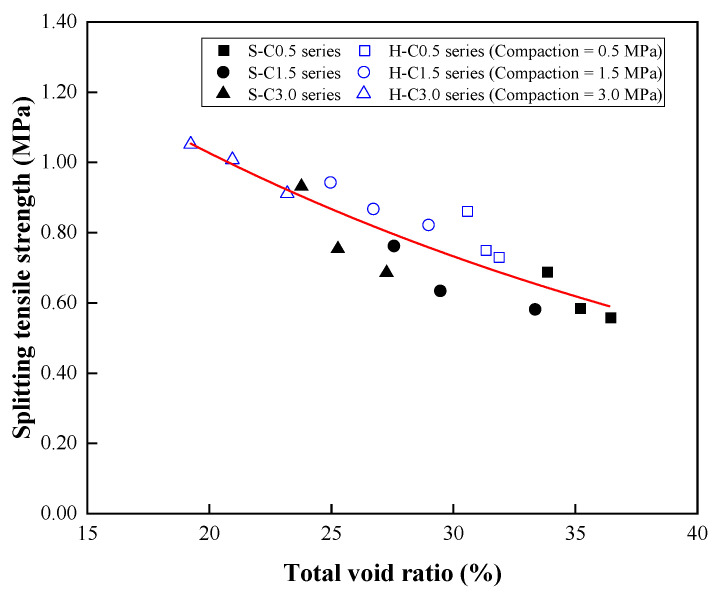
Relationship between the spitting tensile strength and total void ratio.

Figure 14 presents the relationship between the splitting tensile strength and total void ratio of the CBA pervious concrete. The following equation can be used to predict the splitting tensile strength of CBA pervious concrete based on the total void ratio:

(4) $$f_{s}=2.0198e^{-0.0339v}\;\;\;R^{2}=0.76$$

where *f_s_* is the splitting tensile strength (MPa) and *ν* is the total void ratio (%).

**Figure 15:**
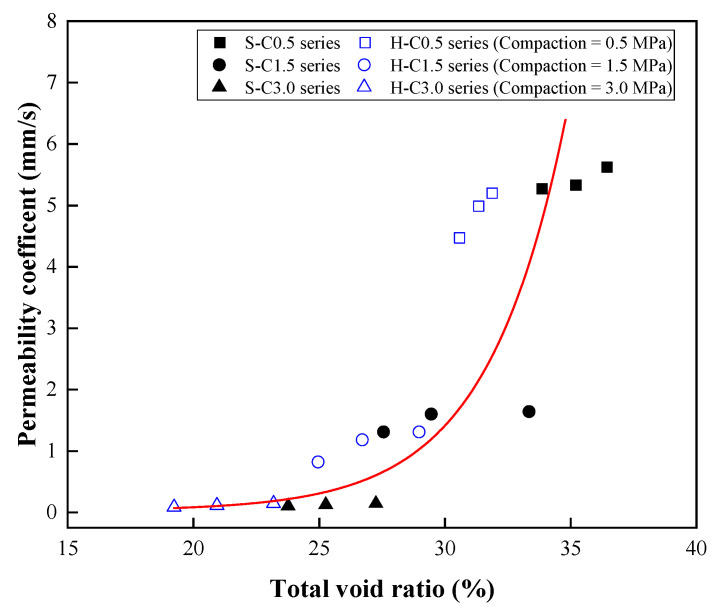
Relationship between the permeability and total void ratio.

Finally, the relationship between the permeability and total void ratio is illustrated in Figure 15. The results show that the permeability and total void ratio had an exponential relationship. The permeability increased with increasing total void ratio. The prediction for the permeability based on the total void ratio was as follows:

(5) $$P_{coef}=0.0001e^{0.3175v}\;\;\;R^{2}=0.77$$

where *P_coef_* is the permeability coefficient (mm/s) and *v* is the total void ratio (%).

The coefficients of determination (*R*^2^) of the three equations in Equation (3) through (5) were greater than 0.7, which implied that the equations could be used to reasonably predict the compressive and splitting tensile strength and the permeability coefficient of the CBA pervious concrete by using the measurement of the total void ratio.

## 6. Conclusions

This study investigated the strength and permeability properties in CBA pervious concrete. In addition, the relationships between the strength properties and total void ratio were presented. The following key findings and conclusions can be drawn from the extensive test results and discussion:

1. By increasing the W/C ratio, the permeability of the CBA pervious concrete slightly increased. In terms of the effect of the compaction level on the total void ratio, the compaction level significantly reduced the permeability of the CBA pervious concrete. At a compaction level of 3.0 MPa, the CBA pervious concrete almost lost its permeability properties. In addition, the use of the hybrid CBA type slightly reduced the permeability of the CBA pervious concrete.

2. The compressive strength of CBA pervious concrete tended to decrease as the W/C ratio increased. The test results revealed that compaction levels had a great effect on the compressive strength of the CBA pervious concrete. In particular, the compaction of the pervious concrete affected the compressive strength of the pervious concrete with the hybrid type of CBA aggregate to a greater degree than that of the pervious concrete with single-type CBA aggregate.

3. An increase in the W/C ratio reduced the splitting tensile strength of the CBA pervious concrete. The compaction levels had a significant effect on the splitting tensile strength of CBA pervious concrete. Specifically, the splitting tensile strength of the pervious concrete with hybrid CBA aggregates improved by 22~33%. Finally, the use of hybrid CBA aggregates was more favorable for improving the splitting tensile strength of CBA pervious concrete.

4. The flexural tensile strength of the pervious concrete with hybrid CBA aggregates decreased by 26~31% as the W/C ratio increased from 0.25 to 0.35. In addition, the flexural tensile strength of the pervious concrete with single-type CBA aggregates increased by 2.0~2.5 times and that of the pervious concrete with hybrid CBA aggregates increased by 1.7~2.0 times as the compaction of the pervious concrete increased from 0.5 to 3.0 MPa.

5. The relationships between the strengths, permeabilities and total void ratios were investigated by regression analyses. Equations from the regression analyses could be reasonably used to predict the strengths and permeabilities of the CBA pervious concrete specimens because the coefficients of determination of the equations were greater than 0.7.

## Figures and Tables

**Table 1:** Physical properties of the aggregates used.

Aggregate Size	Water Absorption	SSD Density	Oven-Dried Density
	(%)	(g/cm^3^)	(g/cm^3^)
2.5–5.0 mm	10.7	1.71	1.54
2.5–5.0 mm (80%) + 1.2–2.5 mm (20%)	7.75	1.73	1.61
1.2~2.5 mm	6.13	1.75	1.65

**Table 2:** Mixing proportions and compaction levels.

Mixture	W/C Ratio	Mixture Proportions (kg/m^3^)				C.A.	Compaction Levels
		Water	OPC	CBA			MPa
				1.2~2.5 mm	2.5~5.0 mm		
S-W25-C0.5	0.25	110.0	440.0	-	1206.1	38.5	0.5
S-W25-C1.5		110.0	440.0	-	1206.1	38.5	1.5
S-W25-C3.0		110.0	440.0	-	1206.1	38.5	3.0
S-W30-C0.5	0.30	110.0	366.7	-	1245.9	38.5	0.5
S-W30-C1.5		110.0	366.7	-	1245.9	38.5	1.5
S-W30-C3.0		110.0	366.7	-	1245.9	38.5	3.0
S-W35-C0.5	0.35	110.0	314.3	-	1274.3	38.5	0.5
S-W35-C1.5		110.0	314.3	-	1274.3	38.5	1.5
S-W35-C3.0		110.0	314.3	-	1274.3	38.5	3.0
H-W25-C0.5	0.25	110.0	440.0	246.9	964.9	38.5	0.5
H-W25-C1.5		110.0	440.0	246.9	964.9	38.5	1.5
H-W25-C3.0		110.0	440.0	246.9	964.9	38.5	3.0
H-W30-C0.5	0.30	110.0	366.7	255.0	996.7	38.5	0.5
H-W30-C1.5		110.0	366.7	255.0	996.7	38.5	1.5
H-W30-C3.0		110.0	366.7	255.0	996.7	38.5	3.0
H-W35-C0.5	0.35	110.0	314.3	260.8	1019.5	38.5	0.5
H-W35-C1.5		110.0	314.3	260.8	1019.5	38.5	1.5
H-W35-C3.0		110.0	314.3	260.8	1019.5	38.5	3.0

Notes: OPC: ordinary Portland cement, CBA: coal bottom ash, and C.A.: cohesive agent.

**Table 3:** Test results of the CBA pervious concrete.

Mixture	Total Void Ratio		Coefficient of Water Permeability		Compressive Strength		Splitting Tensile Strength		Flexural Tensile Strength	
	(%)		(mm/s)		(MPa)		(MPa)		(MPa)	
	Mean	S.D.	Mean	S.D.	Mean	S.D.	Mean	S.D.	Mean	S.D.
S-W25-C0.5	33.9	0.5	5.27	0.21	3.82	0.30	0.69	0.03	0.94	0.20
S-W25-C1.5	27.6	1.1	1.29	0.25	4.38	0.20	0.76	0.04	1.87	0.41
S-W25-C3.0	23.8	0.3	0.08	0.06	5.05	0.30	0.93	0.03	2.34	0.30
S-W30-C0.5	35.2	1.2	5.33	0.33	3.40	0.19	0.58	0.02	0.88	0.35
S-W30-C1.5	29.5	0.8	1.58	0.27	4.19	0.20	0.63	0.04	1.55	0.22
S-W30-C3.0	25.3	0.6	0.1	0.02	4.81	0.45	0.75	0.06	1.85	0.27
S-W35-C0.5	36.5	0.3	5.62	0.25	2.77	0.16	0.56	0.02	0.82	0.06
S-W35-C1.5	33.4	0.5	1.62	0.32	3.16	0.39	0.58	0.03	1.32	0.05
S-W35-C3.0	27.3	0.7	0.12	0.03	3.55	0.41	0.68	0.02	1.68	0.17
H-W25-C0.5	30.6	1.8	4.47	0.61	4.74	0.53	0.86	0.06	1.29	0.12
H-W25-C1.5	24.9	2.8	0.80	0.25	6.37	0.27	0.94	0.04	2.31	0.08
H-W25-C3.0	19.3	2.0	0.06	0.33	7.85	0.87	1.05	0.08	2.70	0.11
H-W30-C0.5	31.3	3.9	5.00	0.51	4.62	0.14	0.75	0.02	1.10	0.01
H-W30-C1.5	26.8	2.8	1.16	0.61	5.94	0.50	0.87	0.01	1.96	0.02
H-W30-C3.0	20.9	1.7	0.09	0.02	7.58	0.52	1.01	0.03	2.01	0.19
H-W35-C0.5	31.9	1.9	5.20	0.06	4.10	0.41	0.73	0.02	1.02	0.09
H-W35-C1.5	29.0	2.0	1.29	0.06	4.90	0.30	0.82	0.05	1.70	0.23
H-W35-C3.0	23.2	3.1	0.12	0.02	5.34	0.42	0.91	0.03	1.79	0.07

## Data Availability

The data used to support the findings in this study are available from the corresponding author upon request.

## References

[1] Abbas S., Arshad U., Abbass W., Nehdi M.L., Ahmed A. (2020). Recycling Untreated Coal Bottom Ash with Added Value for Mitigating Alkali–Silica Reaction in Concrete: A Sustainable Approach. Sustainability.

[2] Kim H.-K. (2022). Coal Bottom Ash. Sustainable Concrete Made with Ashes and Dust from Different Sources.

[3] Al Biajawi M.I., Embong R., Muthusamy K., Ismail N., Obianyo I.I. (2022). Recycled Coal Bottom Ash as Sustainable Materials for Cement Replacement in Cementitious Composites: A Review. Constr. Build. Mater..

[4] Gooi S., Mousa A.A., Kong D. (2020). A Critical Review and Gap Analysis on the Use of Coal Bottom Ash as a Substitute Constituent in Concrete. J. Clean. Prod..

[5] Muthusamy K., Rasid M.H., Jokhio G.A., Mokhtar Albshir Budiea A., Hussin M.W., Mirza J. (2020). Coal Bottom Ash as Sand Replacement in Concrete: A Review. Constr. Build. Mater..

[6] Zaetang Y., Wongsa A., Sata V., Chindaprasirt P. (2015). Use of Coal Ash as Geopolymer Binder and Coarse Aggregate in Pervious Concrete. Constr. Build. Mater..

[7] Mohammed S.A., Koting S., Katman H.Y.B., Babalghaith A.M., Abdul Patah M.F., Ibrahim M.R., Karim M.R. (2021). A Review of the Utilization of Coal Bottom Ash (CBA) in the Construction Industry. Sustainability.

[8] Jeong S.-T., Bui Q.-T., Yang I.-H. (2022). A Comparative Study of the Thermal Conductivities of CBA Porous Concretes. Materials.

[9] Ćosić K., Korat L., Ducman V., Netinger I. (2015). Influence of Aggregate Type and Size on Properties of Pervious Concrete. Constr. Build. Mater..

[10] Strieder H.L., Dutra V.F.P., Graeff Â.G., Núñez W.P., Merten F.R.M. (2022). Performance Evaluation of Pervious Concrete Pavements with Recycled Concrete Aggregate. Constr. Build. Mater..

[11] Park S.B., Il Jang Y., Lee J., Lee B.J. (2009). An Experimental Study on the Hazard Assessment and Mechanical Properties of Porous Concrete Utilizing Coal Bottom Ash Coarse Aggregate in Korea. J. Hazard. Mater..

[12] Kováč M., Sičáková A. (2018). Pervious Concrete as an Environmental Solution for Pavements: Focus on Key Properties. Environments.

[13] Li L.G., Feng J.J., Lu Z.C., Xie H.Z., Xiao B.F., Kwan A.K.H., Jiao C.J. (2022). Effects of Aggregate Bulking and Film Thicknesses on Water Permeability and Strength of Pervious Concrete. Powder Technol..

[14] Pereira da Costa F.B., Haselbach L.M., da Silva Filho L.C.P. (2021). Pervious Concrete for Desired Porosity: Influence of w/c Ratio and a Rheology-Modifying Admixture. Constr. Build. Mater..

[15] Bonicelli A., Giustozzi F., Crispino M. (2015). Experimental Study on the Effects of Fine Sand Addition on Differentially Compacted Pervious Concrete. Constr. Build. Mater..

[16] (2010). Standard Test Method for Density and Absorption of CBA.

[17] Kim H.K., Lee H.K. (2011). Use of Power Plant Bottom Ash as Fine and Coarse Aggregates in High-Strength Concrete. Constr. Build. Mater..

[18] Singh M., Siddique R. (2015). Properties of Concrete Containing High Volumes of Coal Bottom Ash as Fine Aggregate. J. Clean. Prod..

[19] Rafieizonooz M., Mirza J., Salim M.R., Hussin M.W., Khankhaje E. (2016). Investigation of Coal Bottom Ash and Fly Ash in Concrete as Replacement for Sand and Cement. Constr. Build. Mater..

[20] Kim H.K., Jeon J.H., Lee H.K. (2012). Flow, Water Absorption, and Mechanical Characteristics of Normal- and High-Strength Mortar Incorporating Fine Bottom Ash Aggregates. Constr. Build. Mater..

[21] Hashemi S.S.G., Mahmud H.B., Ghuan T.C., Chin A.B., Kuenzel C., Ranjbar N. (2019). Safe Disposal of Coal Bottom Ash by Solidification and Stabilization Techniques. Constr. Build. Mater..

[22] Kearsley E.P., Wainwright P.J. (2001). Porosity and Permeability of Foamed Concrete. Cem. Concr. Res..

[23] (2019). Testing Hardened Concrete—Part 3: Compressive Strength of Test Specimens.

[24] (2019). Testing Hardened Concrete—Part 6: Tensile Splitting Strength of Test Specimens.

[25] (2019). Testing Hardened Concrete—Part 5: Flexural Strength of Test Specimens.

[26] Debnath B., Sarkar P.P. (2019). Permeability Prediction and Pore Structure Feature of Pervious Concrete Using Brick as Aggregate. Constr. Build. Mater..

[27] Zhu H., Wen C., Wang Z., Li L. (2020). Study on the Permeability of Recycled Aggregate Pervious Concrete with Fibers. Materials.

[28] Cui X., Zhang J., Huang D., Liu Z., Hou F., Cui S., Zhang L., Wang Z. (2017). Experimental Study on the Relationship between Permeability and Strength of Pervious Concrete. J. Mater. Civ. Eng..

[29] Torres A., Aguayo F., Gaedicke C., Nerby P., Cavazos M., Nerby C. (2020). Developing High Strength Pervious Concrete Mixtures with Local Materials. J. Mater. Sci. Chem. Eng..

[30] Torres A., Hu J., Ramos A. (2015). The Effect of the Cementitious Paste Thickness on the Performance of Pervious Concrete. Constr. Build. Mater..

[31] Wang Z., Zou D., Liu T., Zhou A. (2021). Influence of Paste Coating Thickness on the Compressive Strength, Permeability, and Mesostructure of Permeable Concrete. Constr. Build. Mater..

[32] Xie X., Zhang T., Yang Y., Lin Z., Wei J., Yu Q. (2018). Maximum Paste Coating Thickness without Voids Clogging of Pervious Concrete and Its Relationship to the Rheological Properties of Cement Paste. Constr. Build. Mater..

[33] Sahdeo S.K., Chandrappa A., Biligiri K.P. (2021). Effect of Compaction Type and Compaction Efforts on Structural and Functional Properties of Pervious Concrete. Trans. Dev. Econ..

[34] Yang L., Kou S., Song X., Lu M., Wang Q. (2021). Analysis of Properties of Pervious Concrete Prepared with Difference Paste-Coated Recycled Aggregate. Constr. Build. Mater..

[35] Nguyen D.H., Sebaibi N., Boutouil M., Leleyter L., Baraud F. (2014). A Modified Method for the Design of Pervious Concrete Mix. Constr. Build. Mater..

